# Linking Hospitalized Patients With Opioid Use Disorder to Treatment—The Importance of Care Transitions

**DOI:** 10.1001/jamanetworkopen.2023.56382

**Published:** 2024-02-05

**Authors:** Marlene Martin, Noa Krawczyk

**Affiliations:** Department of Medicine, Division of Hospital Medicine, University of California, San Francisco and San Francisco General Hospital; Department of Population Health, Center for Opioid Epidemiology and Policy (COEP), NYU Grossman School of Medicine, New York

Hospitalizations involving patients with opioid use disorder (OUD) result in substantial costs and burdens on health systems and are increasing in the setting of the current US overdose crisis. Multiple interventions to improve care for hospitalized patients with OUD have arisen, including addiction consult teams (ACTs), practice-based OUD treatment models, and linkage supports (eg, peer support specialists, patient navigators, social workers).^1^ Although hospitalized patients with OUD may not necessarily be seeking treatment for OUD during hospitalization, they are often receptive to treatment. Offering medication for OUD (MOUD) during hospitalization increases MOUD initiation rates, reduces self-directed discharges, and improves the care experience. To continue the benefits of OUD treatment after hospitalization, hospitals must ensure seamless care transitions for patients to access ongoing outpatient care. This is critical given the nearly 8% mortality rate among hospitalized patients with OUD within the first year after hospital discharge and because MOUD reduces both all-cause and overdose mortality. Thus, efforts to improve care transitions and specifically linkage to postdischarge MOUD are important.

The study by Marcovitz and colleagues^2^ is a pragmatic, randomized trial in a tertiary care hospital with an ACT and its colocated hospital bridge clinic. All patients with OUD without an MOUD clinician seen by the hospital’s ACT who accepted buprenorphine or injectable naltrexone were randomized to bridge clinic or usual care. The bridge clinic group received enhanced case management during hospitalization, an MOUD prescription at discharge, and referral to the colocated bridge clinic. The usual care group also received an MOUD prescription at discharge and was referred to a community MOUD clinic. The authors found that median length of stay, the primary outcome, did not differ between the groups and at 16 weeks, those referred to the bridge clinic had fewer hospital-free days, more readmissions to the study hospital, and higher care costs. There were no differences in emergency department visits or deaths between groups. However, those in the bridge clinic group were more likely to receive self-reported MOUD clinician linkage, had more MOUD refills, and were less likely to experience an overdose.

Although the authors did not find that referral to the hospital’s colocated bridge clinic reduced hospitalization length, it did improve multiple key outcomes over the 16-week follow-up, including increased number of buprenorphine fills, which reduces overdose risk and improves other health outcomes. The literature surrounding best practices in care transitions for hospitalized patients with OUD is limited but of growing interest as hospitals seek to address the overdose crisis. A recent scoping review^3^ of substance use–related care transition strategies from acute care to outpatient settings found the predischarge strategies most commonly described included scheduling an appointment, discussing treatment options, unspecified linkage to treatment, and giving patients a clinician list. Postdischarge strategies commonly described included transportation assistance and care navigation, bridge prescription and follow-up calls and/or texts, and peer support.^3^ In the study by Marcovitz et al,^2^ both the bridge clinic and usual care groups received many of the care transition strategies highlighted by this scoping review, with the main difference between intervention groups being the type of predischarge linkage to treatment. Patients in the bridge clinic group received enhanced case management during hospitalization and met bridge staff prior to discharge. These patients were more likely to link to the bridge clinic, had more MOUD refills, and were less likely to experience an overdose—likely due to MOUD’s life-saving impact.

Marcovitz et al^2^ found that there were increased costs and more readmissions to the study hospital in the bridge clinic group in the short term. However, this may result from patients previously underutilizing care; it is possible that short term increased acute care utilization and costs may decrease overall long-term acute care utilization, health care costs, and mortality. This finding may also reflect increased trust in the health care system associated with the bridge clinic group, as stigma often prevents patients with OUD from seeking and accessing care. A qualitative study that examined the perspectives of community clinicians and staff toward buprenorphine initiated in the emergency department found that barriers to successful OUD treatment linkage included negative attitudes about MOUD, limited treatment capacity, the reported need for specialized addiction clinicians, and addressing the social determinants of health to support treatment engagement.^4^ Bridge clinics address many of these while providing stigma-free, low-barrier, and expert addiction care. However, bridge clinics alone cannot care for the many patients with OUD, just as not all patients with diabetes can or need to be seen in endocrinology clinic. It is important to recognize that while many patients with OUD may benefit from bridge clinic care at least transiently, community clinics will also need to build OUD treatment capacity. Many opportunities remain for hospital systems to partner with community clinics and clinicians, harm reduction services, and organizations that address the social determinants of health that may further enhance linkage and retention in OUD treatment.

To effectively leverage the opportunity that hospitalization presents to offer and initiate MOUD, further efforts must elucidate the best evidence-based practices for care transitions. A recent national study of Community Health Needs Assessment Reports of nonprofit hospitals found that only 17% offered acute care interventions to support patients’ transition to community-based addiction services, with even fewer providing detail on the strategies used to do so.^5^ We have a unique opportunity to expand use of and linkage to outpatient MOUD following the Consolidated Appropriations Act of 2023, which removed the X waiver required to prescribe buprenorphine among discharging hospital-based clinicians and outpatient clinicians. We need studies to assess how this is affecting initiation and linkage to buprenorphine among hospitalized patients and examine remaining barriers for successful engagement and retention in care. The Drug Enforcement Agency (DEA) has also tried to increase access to MOUD by allowing DEA-registered practitioners in acute care settings to request an exception to 21 CFR 1306.97(b) and dispense up to 3 days of methadone at discharge.^6^ However, impact is limited because some state regulations restrict implementation. Another effort to expand access to methadone is the Modernizing Opioid Treatment Access Act, which if passed, will increase access to methadone by allowing board certified addiction specialists to prescribe methadone outside of more restricted opioid treatment programs.

Given the large treatment gap that remains, with only 13% of patients with OUD receiving life-saving MOUD, improving initiation, linkage, and ongoing retention while increasing accessibility to MOUD and substance use disorder treatment more broadly remains a crucial challenge and goal for the field of addiction medicine.^7^ While bridge clinics may present a promising care transition strategy to help engage patients with OUD after hospital discharge care, we need to understand the impact of such clinics on long-term patient outcomes and how to adapt health care services to maximize treatment retention both in specialty addiction care and general medical clinics so that all doors offer accessible treatment while reducing costs for health systems and payers.

## References

[1] James H, Morgan J, Ti L, Nolan S. Transitions in care between hospital and community settings for individuals with a substance use disorder: a systematic review. Drug Alcohol Depend. 2023;243:109763. doi:10.1016/j.drugalcdep.2023.109763 36634575

[2] Marcovitz D, Dear ML, Donald R, Effect of a co-located bridging recovery initiative on hospital length of stay among patients with opioid use disorder: the BRIDGE randomized clinical trial. JAMA Netw Open. 2024;7(2):e2356430. doi:10.1001/jamanetworkopen.2023.56430 38411964 PMC10900965

[3] Krawczyk N, Rivera BD, Chang JE, Strategies to support substance use disorder care transitions from acute-care to community-based settings: a scoping review and typology. Addict Sci Clin Pract. 2023;18(1):67. doi:10.1186/s13722-023-00422-w 37919755 PMC10621088

[4] Sue KL, Chawarski M, Curry L, Perspectives of clinicians and staff at community-based opioid use disorder treatment settings on linkages with emergency department-initiated buprenorphine programs. JAMA Netw Open. 2023;6(5):e2312718. doi:10.1001/jamanetworkopen.2023.12718 37163263 PMC10173026

[5] Krawczyk N, Rivera BD, Chang JE, Lindenfeld Z, Franz B. Initiatives to support the transition of patients with substance use disorders from acute care to community-based services among a national sample of nonprofit hospitals. J Addict Med. 2023. doi:10.1097/ADM.0000000000001250 PMC10939963 38015653

[6] Skogrand E, Sharpe J, Englander H. Dispensing methadone at hospital discharge: one hospital’s approach to implementing the “72-hour rule” change. J Addict Med. 2023. doi:10.1097/ADM.0000000000001246 PMC10873107 37994453

[7] Krawczyk N, Rivera BD, Jent V, Keyes KM, Jones CM, Cerdá M. Has the treatment gap for opioid use disorder narrowed in the US?: a yearly assessment from 2010 to 2019”. Int J Drug Policy. 2022;110:103786. doi:10.1016/j.drugpo.2022.103786 35934583 PMC10976290

